# Spectroscopic Studies of Mononuclear Molybdenum Enzyme Centers

**DOI:** 10.3390/molecules27154802

**Published:** 2022-07-27

**Authors:** Martin L. Kirk, Russ Hille

**Affiliations:** 1 Department of Chemistry and Chemical Biology, The University of New Mexico, MSC03 2060, 1 University of New Mexico, Albuquerque, NM 87131-0001, USA; 2 Department of Biochemistry, Boyce Hall 1463, University of California, Riverside, CA 82521, USA

**Keywords:** xanthine oxidase, sulfite oxidase, DMSO reductase, electronic absorption spectroscopy, EPR spectroscopy, X-ray absorption spectroscopy, resonance Raman spectroscopy, magnetic circular dichroism spectroscopy

## Abstract

A concise review is provided of the contributions that various spectroscopic methods have made to our understanding of the physical and electronic structures of mononuclear molybdenum enzymes. Contributions to our understanding of the structure and function of each of the major families of these enzymes is considered, providing a perspective on how spectroscopy has impacted the field.

## 1. Introduction

**Figure 1:**
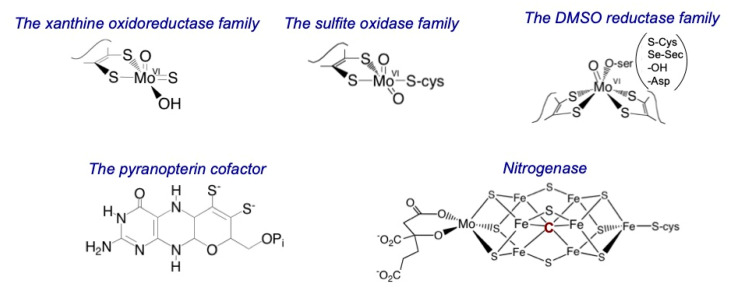
The families of molybdenum-containing enzymes.

A variety of spectroscopic methods have provided critical information regarding the physical and electronic structures of the active sites of mononuclear molybdenum-containing enzymes, ranging in sophistication from standard ultraviolet (UV)/visible absorption spectroscopy and electron paramagnetic resonance (EPR) to more sophisticated methods such as X-ray absorption, resonance Raman and magnetic circular dichroism spectroscopies [1,2]. We consider here each of these methods in turn as they have been applied to each of the major families of molybdenum-containing enzymes, with an emphasis on the type of information obtained by each technique. The oxidized active site structures for each of these families are shown in Figure 1. Briefly, the molybdenum centers of the xanthine oxidase family can be formulated as LMo^VI^OS(OH) (L = pyranopterin dithiolene, PDT) in a square-pyramidal coordination geometry with an apical oxo ligand (Mo≡O) and an equatorial sulfido (Mo=S). Molybdenum centers of the sulfite oxidase (SO) family are formulated as LMo^VI^O_2_(S-Cys); the coordination geometry is again square-pyramidal with apical and equatorial terminal oxo donors, but the stereochemistry is opposite that seen in the xanthine oxidase family. The molybdenum centers seen in members of the dimethylsulfoxide (DMSO) reductase family are L_2_Mo^VI^XY, in a distorted trigonal prismatic coordination geometry, with X being either a Mo≡O or Mo≡S group; Y can be a Mo-OH, O-Ser, S-Cys, Se-Sec, or Asp ligand, depending on the protein. Note that when only a single teminal oxo (or sulfido) ligand is present, the M-E bond order is three, with one σ and two π interactions between the ligand and metal. Due to space limitations, the complex multinuclear molybdenum center of nitrogenase is not considered here.

## 2. Electronic Absorption Spectroscopy

Electronic absorption spectroscopy is one of the most widely applied spectroscopic methods in studies of molybdenum-containing enzymes. While, as discussed further below, the molybdenum center itself often contributes only minimally to the overall absorption spectrum of oxidized enzyme or to the spectral change seen upon reduction, it nevertheless has been an essential tool used to understand the behavior of these enzymes in the course of equilibrium reductive titrations and rapid-reaction kinetic studies due to the much larger spectral contributions of other redox-active centers that typically accompany the molybdenum centers in these enzymes.

### 2.1. Xanthine Oxidase and Related Enzymes

Members of the xanthine oxidase family of enzymes have, minimally, a pair of [2Fe-2S] iron-sulfur clusters in each subunit of the enzyme (the enzymes being most often organized as homodimers); most also possess one equivalent of flavin adenine dinucleotide (FAD) as well. The contributions of the [2Fe-2S] and FAD centers dominate the electronic absorption spectrum of these enzyme and do not report directly on their molybdenum centers. Most members of this enzyme family function as hydroxylases with the substrate that becomes oxidized (e.g., xanthine) introducing pairs of reducing equivalents into the enzyme at its molybdenum center. Subsequent sequential transfer of the individual reducing equivalents out of the molybdenum center to the other redox-active centers of the enzyme provides the spectral changes that make it possible to follow, for example, the kinetics of the overall reaction. Rapid reaction kinetics of these enzymes following UV/visible spectral changes have in fact been critical to our understanding of the reactions catalyzed by these enzymes. Kinetic analysis is generally simplified by the fact that intramolecular electron transfer is invariably very rapid compared to the chemistry taking place at the molybdenum center [3,4,5], but it must always be kept in mind in such studies that the nature of the substrate transformation and subsequent electron transfers are in fact discrete processes and the observed spectral changes only indirectly probe the molybdenum center.

Nevertheless, the use of electronic absorption spectroscopy to follow the molybdenum center directly has been successfully applied in specific cases. Using the slow substrate 2-hydroxy-6-methyl-purine, McWhirter and Hille [6] were able to demonstrate that reduction of the molybdenum center of bovine xanthine oxidase from the oxidized Mo^VI^ state to the Mo^IV^ state with bound product yielded a spectral change with an absorbance minimum at 370 nm and maximum at 470 nm. Oxidation to the Mo^V^ state (with product still bound, as determined by EPR, see below) caused a spectral shift with a minimum at 420 nm and maximum at 540 nm, with further oxidation returning the molybdenum center to the oxidized Mo^VI^ state. The extinction changes for both intermediate species were very small, less than 1.0 mM^−1^cm^−1^. Quantification of the reducing equivalents leaving the enzyme (as superoxide, as detected by the reduction of cytochrome *c*) demonstrated that generation of the 540 nm-absorbing species resulted in the loss of a first electron from the enzyme and its decay to oxidized enzyme a second, thus demonstrating that the generation of the Mo^V^ state was an oxidative event, implying in turn that the initial reduction was a two-electron event that took the molybdenum center directly from Mo^VI^ to Mo^IV^. Subsequent work by Ryan et al. [7] determined the spectral change associated with reduction of the molybdenum center in bovine xanthine oxidase (and chicken liver xanthine dehydrogenase) by sodium dithionite to have a maximum absorption change at 430 nm, with the magnitude of the absorbance change approximately tripling over the pH range 6 to 10. This spectral change was again very weak, with an estimated extinction change of 2.4 mM^−1^cm^−1^ for xanthine oxidase, and 1.2 mM^−1^cm^−1^ for xanthine dehydrogenase. Lumazine [8,9,10], 4-thiolumazine [11,12,13], and 2,4-dithiolumazine [11,12,13] have also been used as substrates for bovine xanthine oxidase and bacterial xanthine dehydrogenase. Their two-electron reduction to the corresponding violopterin products leads to the formation of stable Mo(IV)-product complexes where the product is bound to Mo(IV) as the enolate tautomer through an Mo-O-C_product_ bridge. For Mo(IV)-thioviolapterin and Mo(IV)-dithioviolapterin, the single intense long wavelength absorption feature observed in the Mo(IV)-violapterin complex is shifted to the near-infrared (NIR) region of the spectrum (758–778 nm) [11,12,13]. Neither the band energy or the overall band shape of the Mo(IV) → product charge transfer feature changed between the wild-type bacterial dehydrogenase and its Q197A and Q102G variants. This observation indicates that Q197 and Q102 do not affect the nature of Mo-product bonding in these Mo(IV)-product complexes. The nature of the NIR charge-transfer transitions in these Mo(IV)−P complexes have been probed by CASSCF calculations, which confirm the assignment as a metal-to-ligand charge-transfer transition (MLCT), originating from a Mo(xy) → product(π*) one-electron promotion [12].

### 2.2. Sulfite Oxidase and Related Enzymes

The longest studied sulfite oxidases have been from vertebrate sources such as chicken liver, and like the xanthine oxidase family these possess an additional site in addition to the molybdenum center (which is again the site of enzyme reduction by substrate)—in this case a strongly absorbing *b*-type cytochrome. Again, UV/visible spectroscopy has been very useful in following the rapid reaction kinetics of the system as the heme becomes reduced, but as indicated above these studies do not directly track the chemistry taking place at the molybdenum center. To avoid difficulties associated with the strongly absorbing heme, the molybdenum-containing domain of human sulfite oxidase has been cloned and over-expressed [14]. The oxidized fragment has absorption maxima at 490 and 360 nm, and the reduced fragment has a single absorption maximum at ~390 nm (although the extinction coefficients were not reported in this work).

Unlike the vertebrate sulfite oxidases, the sulfite oxidase from *A. thaliana* is devoid of additional redox-active centers and this has permitted the characterization of the absorption properties of its molybdenum center. The oxidized enzyme exhibits increasing absorption going to shorter wavelengths, with shoulders at 470 nm and 360 nm, and the sulfite-reduced enzyme has a single broad shoulder centered at ~390 nm [15], all features shared with the molybdenum-containing fragment of human sulfite oxidase [14]. The molybdenum-containing domain of human sulfite oxidase has been cloned and expressed, and found to have very similar absorption spectra to the *A. thaliana* enzyme [16]; surprisingly, mutation of the metal-coordinating cysteine to selenocysteine in the human molybdenum domain does not significantly perturb the absorption features of the domain. The absorption features are shifted to somewhat longer wavelength in the case of the molybdenum-containing component of the human mitochondrial amidoxime reducing complex (hmARC), another member of the sulfite oxidase family that lack sites other than the molybdenum center [17]. The absorption features of both the plant and human proteins are very weak, with extinction coefficients of approximately the same magnitude as those seen with xanthine oxidase above (i.e., 1–2 mM^−1^cm^−1^).

A final member of the sulfite oxidase family, again devoid of additional redox-active centers is the *E. coli* methionine sulfoxide reductase (product of the *yedY* gene). The as-isolated enzyme, in the Mo^V^ state, exhibits multiple weak absorption bands with an extinction maximum of ~1.5 mM^−1^cm^−1^ at 360 nm (reported as 28,000 cm^−1^) [18]. As discussed further below, more recent work has suggested that the as-isolated enzyme is found in complex with an unknown sulfur-containing inhibitor coordinated to the Mo ion [19].

### 2.3. DMSO Reductase and Related Enzymes

**Figure 2:**
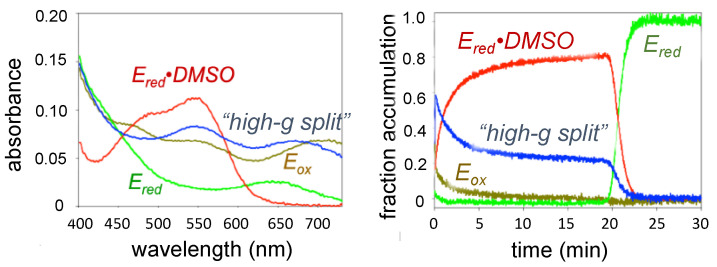
UV/visible absorption spectra of catalytic intermediates for DMSO reductase (**left**) and their time courses (**right**) seen in the course of turnover with DMSO as substrate. E_ox_, the Mo(V) species giving rise to the “high-g split” EPR signal, E_red_ and E_red_•DMSO are color-coded for clarity.

The DMSO reductase family of molybdenum-containing enzymes possess molybdenum centers that are by far the most strongly absorbing in the UV/visible, although generalizations regarding their spectroscopic properties is complicated by the ligand diversity of the family. Nevertheless, some important results have been forthcoming. The DMSO reductase from organisms such as *Rhodobacter sphaeroides* possess an L_2_MoO(O-Ser) active site as their sole redox-active center. As shown in Figure 2, the oxidized enzyme absorbs throughout the UV/visible, with absorption maxima at 720 nm and 360 nm along with shoulders at 560 nm and 460 nm. The dithionite-reduced enzyme has maxima at 640 nm and 380 nm, and a complex of reduced enzyme in complex with DMSO an absorption maximum at 560 nm with a shoulder at 480 nm. The extinction coefficients for all three species are in the 2–4 mM^−1^cm^−1^ range. These spectral properties have been exploited to undertake enzyme-monitored turnover experiments in which enzyme at a concentration sufficient to follow its absorption is monitored over time as the enzyme turns over with either DMSO or trimethylamine-N-oxide (another substrate for the enzyme), using sodium dithionite as (non-physiological) reductant [20]. In addition to E_ox_, E_red_ and E_red_•DMSO, a fourth species is observed with wavelength maxima at 670, 570 and 380 nm. This species accumulates to ~20% of the total enzyme during turnover with DMSO, but to essentially 100% (and over an extended period of time) with trimethylamine-N-oxide (TMAO) as substrate. It was shown that this species corresponds to that giving rise to the so-called “high-g split” EPR signal (see below), demonstrating that this species was indeed catalytically relevant, as had been proposed previously by Bray and coworkers [21,22]. The electronic absorption spectrum of “high-g split” has been studied in detail, and this has resulted in a complete assignment of the observed magnetic circular dichroism (MCD) and electronic absorption bands (again, see below) [23]. This work has provided a deep understanding of the metal-ligand bonding scheme in this intermediate that has provided key information regarding the molybdenum coordination geometry and coordination number, and Mo-S covalency contributions to oxygen atom transfer and electron transfer reactivity [23]. Unfortunately, electronic absorption spectroscopy has proven less generally useful in studies of the molybdenum centers of most other members of the DMSO family owing to the presence again of multiple redox-active centers whose absorption properties obscure those of the molybdenum center.

## 3. EPR and Related Methods

EPR, probing the S = 1/2 Mo(V) oxidation state, has been one of the principal tools to study molybdenum-containing enzymes for over 50 years, beginning with the demonstration in 1966 by Bray and Meriwether [24] that bovine xanthine oxidase possessed molybdenum in its active site, and that the metal became reduced in the course of the reaction of enzyme with substrate. Since then, major advances to our understanding of the active sites of each family of molybdenum-containing enzyme have been made using EPR.

### 3.1. Xanthine Oxidase Family Enzymes

**Figure 3:**
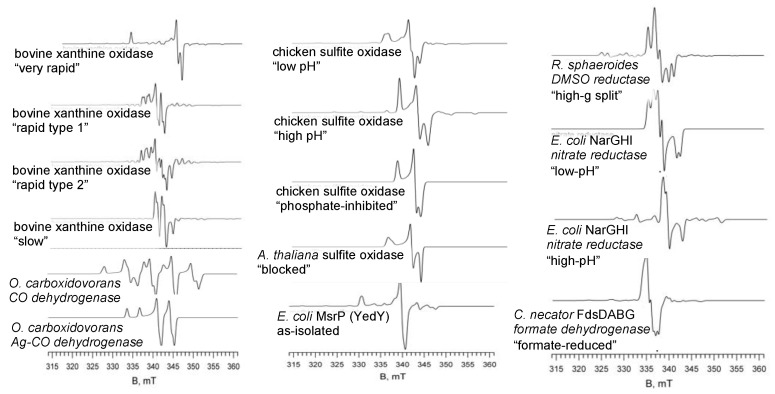
EPR signals seen with various molybdenum-containing enzymes. (**Left**), the “Very Rapid”, “Rapid Type 1”, “Rapid Type 2” and “Slow” signals of xanthine oxidase, the Mo/Cu center of CO dehydrogenase and its Ag-substituted form. (**Center**), the “Low-pH”, “High-pH” and “Phosphate-inhibited” forms of chicken sulfite oxidase, the “Blocked” signal seen with the *A. thaliana* enzyme and the EPR signal seen with as-isolated MsrP. (**Right**), the “high-g Split” signal seen with *R. sphaeroides* DMSO reductase, low- and high-pH forms of *E. coli* nitrate reductase and *C. necator* formate dehydrogenase. All spectra are simulations at 9 GHz using published g-values and hyperfine couplings (see text).

Following on the initial discovery of molybdenum in xanthine oxidase, a freeze-quench protocol was developed [25,26] that identified, in addition to EPR signals of the iron-sulfur clusters and FADH• of the enzyme, two discrete Mo(V) EPR signals were identified that were ultimately termed “Rapid” and “Very Rapid” [27]. The “Very Rapid” signal was nearly axial in lineshape and was devoid of proton hyperfine, while the “Rapid” signal was more uniformly rhombic and exhibited coupling to two inequivalent protons (one strongly coupled, the second more weakly). The strongly coupled proton in the “Rapid” species was found to arise from the C-8 position of xanthine, the position hydroxylated in the course of oxidation of xanthine to uric acid [28]. A fourth “Slow” form was subsequently found to arise from nonfunctional enzyme that had lost the catalytically essential Mo=S, being replaced by a second Mo=O [27]. These signals and others from different molybdenum containing enzymes are shown in Figure 3.

**Figure 4:**
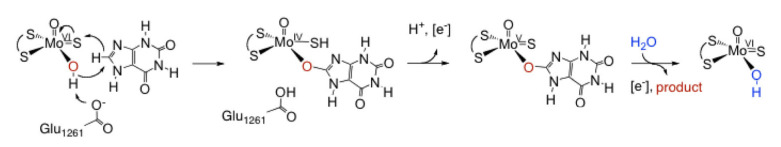
The reaction mechanism of xanthine oxidase.

The relative amounts of “Very Rapid” and “Rapid” signals varied with the reaction conditions and substrate used. 2-hydroxy-6-methylpurine formed especially high levels of the species giving rise to the “Very Rapid” signal [29], which was understood to arise from a catalytic intermediate. Electron spin echo envelope modulation (ESEEM) work tracking weak ^1^H coupling [30], electron nuclear double resonance (ENDOR) studies with 8-^13^C-2-hydroxy-6-methylpurine [31] and ultimately protein crystallography [32] established that the species giving rise to the “Very Rapid” EPR signal had the 8-hydroxylated product coordinated equatorially to the molybdenum via the catalytically introduced hydroxyl group in a simple end-on fashion. It had been shown in earlier work that the molybdenum coordination sphere possessed a catalytically labile oxygen [33], and additional EPR work with ^17^O demonstrated that this was the equatorial Mo-OH group of the oxidized enzyme [34]. It is now generally understood that the reaction proceeds by base-assisted nucleophilic attack of the equatorial Mo-OH of the molybdenum center on the C-8 position of substrate, with concomitant hydride transfer to the M=S as shown in Figure 4. The closely related aldehyde oxidases, which share considerable overlap in substrate specificity with the xanthine oxidases, are considered to operate similarly.

No “Rapid” signal is seen in the course of the reaction of enzyme with substoichiometric 2-hydroxy-6-methylpurine, and subsequent work showed that this signal simply arose from binding of substrate to enzyme that had been partially reduced by reaction with a prior equivalent of substrate, with the molybdenum center in the Mo(V) state [35]. The signal-giving species was thus formally a dead-end intermediate but, importantly, represented a paramagnetic analog of the Michaelis complex. Paradoxically, the species giving rise to the “Rapid” signal therefore lies upstream rather than downstream to that giving rise to the “Very Rapid” EPR signal, and in fact partial reduction of the enzyme prior to mixing with the slow substrate 2-hydroxy-6-methylpurine results in extremely rapid appearance of the “Rapid” signal that precedes the eventual accumulation of the “Very Rapid” signal. 

In addition to the above EPR signals relevant to the reaction mechanism of xanthine oxidase, a wide variety of Mo(V) signals arising from nonfunctional forms of the enzyme under various conditions have been observed. These include the “Slow” signal mentioned above, due to the so-called desulfo form of the enzyme in which the catalytically essential equatorial Mo=S has been lost and replaced with a second Mo=O, as well as inhibited forms in complex with alloxanthine, formaldehyde, ethylene glycol, p-chloromercuribenzoate, formamide and arsenite. Detailed EPR and ENDOR studies of the aldehyde-inhibited form of xanthine oxidase have been used to define the geometric and electronic structure of this species [36,37]. The work provided a valence bond description of the xanthine oxidase-mediated C-H bond activation as deriving from a combination of Mo=S π → C-H σ* and C-H σ → Mo=S π* charge transfers in a Woodward-Hoffmann manner, with other charge-transfer energy stabilization being present in the transition state [36]. The EPR signals of these latter species have been summarized elsewhere [38] and are not considered further here.

The active site of CO dehydrogenase from aerobes such as *Oligotropha carboxydovorans* [39,40] is a unique variation on that of other xanthine oxidase family members in that it possesses a binuclear Mo-S-Cu active site, the bridging sulfur occupying the position of the equatorial Mo=S group of other enzyme family members [41]. One-electron reduction yields an EPR signal that is nominally due to Mo(V) (see Figure 3, lower left), but with extremely strong coupling to the ^63/65^Cu nucleus [42]; by analogy to the EPR of binuclear Mo/Cu models [43], the strength of this coupling is taken to reflect considerable spin delocalization onto the copper. ENDOR work with ^13^CO has demonstrated CO binding to the copper [44], suggesting a mechanism in which CO is activated by binding to the Cu of the binuclear center, and with the C-O bond weakened by back-bonding from the copper it is then attacked by the equatorial Mo-OH. Mechanisms that involve bicarbonate as an intermediate are unlikely, as bicarbonate binding to enzyme partially reduced with dithionite yields an EPR signal that is distinct from that seen upon reduction of enzyme with CO [45]. 

As-isolated CODH is often partially depleted of Cu and inactive, but a protocol has been developed utilizing Cu(I) that enables reconstitution of the binuclear center [46]. Interestingly, activity is also recovered when Ag(I) is substituted for the copper in the reconstitution, although the silver-substituted enzyme reacts with CO at only 1/6 the rate of native, copper-containing enzyme [47]. The lower activity is readily rationalized by the known lower backbonding capacity of Ag relative to Cu, which would render the coordinated CO less susceptible to nucleophilic attack in the silver-substituted enzyme. The EPR signal seen with this silver-substituted enzyme exhibits strong hyperfine coupling to Ag (I = ½ for ^107/109^Ag) analogous to that seen with copper in the native enzyme, as shown in Figure 3, bottom left [47].

### 3.2. III.B. Sulfite Oxidase Family Enzymes

The initial EPR work with sulfite oxidase identified three discrete Mo(V) signals using continuous-wave EPR, designated “high-pH”, “low-pH” and “phosphate-inhibited” [48,49], with the “low-pH” form being distinguished by strong coupling to one solvent-exchangeable proton of an equatorial Mo-OH group. The “high-pH” form does in fact exhibit weak proton coupling as manifested in more sensitive pulsed EPR methods [50,51], however, with the weaker coupling being due to rotation of the Mo-OH bond out of the metal xy plane (the redox-active orbital being d_xy_) [52,53]. Phosphate displaces the Mo-OH, and while no ^31^P coupling is seen by the continuous-wave EPR with ^31^P-labeled phosphate, coupling of 4–11 MHz is seen in ESEEM experiments [54]. Chloride also inhibits sulfite oxidase by binding in the substrate binding site and perturbs the ”low-pH” EPR spectrum of sulfite oxidase [55,56]. The spectrum exhibited by the *Arabidopsis thaliana* enzyme has a g-tensor that resembles the canonical “low-pH” signal but lacks ^1^H hyperfine coupling [57,58]. On the basis of the large quadrupole coupling observed by pulsed EPR (36–40 MHz), this signal has been attributed to a complex with sulfite rather than sulfate [52]. The Mo(V) signals of several enzymes of the sulfite oxidase family are shown in Figure 3, center.

Bacterial sulfite-oxidizing enzymes from organisms such as *Starkeya novella* exhibit signals similar to the vertebrate sulfite oxidases [59,60,61], as do the assimilatory nitrate reductases from algae and higher plants [62,63] and the molybdenum-containing components of the hmARC systems [17,64,65]. A combination of computational and EPR studies on mARC have provided information regarding the nature of the reaction coordinate for the one-electron reduction of nitrite to NO [65]. The EPR spectrum of the Mo(V) species generated during this reaction sequence is similar to that of low-pH sulfite oxidase, and the work led to reaction mechanisms being proposed for nitrate reduction. Interestingly, it appears that only the Mo(IV) form of the enzyme, and not the Mo(V) form, is capable of promoting catalytic nitrate reduction in a one-electron process [65]. The X-ray structure of *E. coli* MsrP [66] resembles that expected for reduced sulfite oxidase family enzymes, with an apical oxo, two sulfur donors from the PDT, and a coordinated cysteine thiolate. Interestingly, the electron density in the region of the fourth equatorial is modeled as a coordinated water or hydroxide ligand, with a urea molecule located in the substrate access channel. The EPR of the MsrP methionine sulfoxide reductase, on the other hand, is more complex, and is not related to any previously observed spectra for sulfite oxidase family enzymes (Figure 3, bottom center). The as-isolated protein exhibits a nearly axial EPR signal with no coupling to protons [18]. A comparison of the EPR and X-ray absorption spectroscopy (XAS) of MsrP with suitable model compounds has concluded that the as-isolated enzyme does not have the canonical MoO_2_ structure of the sulfite oxidase family, but instead has an unidentified sulfur-containing inhibitor bound to the molybdenum displacing the equatorial Mo-OH [19]. 

### 3.3. III.C. DMSO Reductase Family Enzymes

The DMSO reductase family is the most diverse of the molybdenum-containing enzymes, and it is not surprising that the EPR properties of family members are equally diverse. The eponymous DMSO reductases from *R. sphaeroides* or *R. capsulatus* are the simplest members of this family [67,68,69], the molybdenum center being their sole redox-active site. No fewer than five EPR signals were initially identified [21], of which that designated “high-g split”, with coupling to one solvent-exchangeable proton, was considered to represent the most mechanistically relevant (a conclusion supported by the enzyme-monitored turnover experiments with the *R. sphaeroides* enzyme described above [20]). The “high-g split” EPR signal has been analyzed in terms of rhombic g- and A-tensors, which are consistent with a stable, low-symmetry 6-coordinate species that possesses a coordination geometry intermediate between idealized octahedral and trigonal prismatic [23]. Interestingly, the spin-Hamiltonian parameters extracted from the data indicate that the singly occupied redox-active orbital is primarily metal ion in character (~77% Mo), with implications for electron transfer and atom transfer reactivity [23]. The “high-g split” signal of DMSO reductase and the Mo(V) signals seen with several other members of the DMSO reductase family are shown in Figure 3, right.

The bacterial nitrate reductases that serve a generally dissimilatory role (i.e., use nitrate as a terminal electron sink) are also members of the DMSO reductase family, of which there are four different types: the membrane-integral and dimeric NarGHI and NarYWV systems [70], the periplasmic Nap protein (NapA or NapAB, depending on the source) [71,72]) and the cytosolic NasA (or NasAB) enzymes [73]. The nitrate reductases differ from the DMSO reductases described above in having aspartate (e.g., in the *E. coli* NarGHI enzyme) or cysteine (e.g., in the periplasmic Nap enzymes from a variety of species) rather than serine coordinated to the molybdenum. The Nap proteins (and possibly the others) also have a terminal Mo=S rather than Mo=O. Like the *R. sphaeroides* DMSO reductase, a variety of EPR signals have been identified in the nitrate reductases from different bacteria, and in the presence of halide inhibitors [74,75,76,77]. It appears that the “high-g split” signal seen with the periplasmic Nap arises from the most directly catalytically relevant form of the enzyme and arises from a species in which the terminal Mo=S is partially bonded to the sulfur of the metal-bond cysteine [78], with the “very high g” signal arising from reduction of the S-S bond to yield terminal Mo=S and Cys thiolate ligands to the molybdenum. Finally, it has been suggested that the first coordination sphere of recombinant *E. coli* TMAO was dependent on the expression conditions. When enzyme was expressed aerobically, the structure was described as [(PDT)_2_MoO(O-Ser)]^1−^, but when expressed anaerobically the site possessed a terminal sulfido ligand instead (i.e., [(PDT)_2_MoS(O-Ser)]^1−^); the extent of Mo≡S formation was reported to correlate with activity. A terminal sulfido ligand was deduced from EPR spectra of enzyme reconstituted with a bis-MGD cofactor from the *R. capsulatus* formate dehydrogenase that could be inserted into the apo-enzyme with the Mo≡S moiety intact. The EPR spectrum of this *E. coli* TMAO lacks proton hyperfine and possesses g-values, determined from spectral simulations, of g_1_ = 1.997, g_2_ = 1.986, g_3_ = 1.967. However, these g-values are very similar to those of other members of the DMSO reductase family that possess a [(PDT)_2_Mo(OH)(O-Ser)]^1−^ structure in the Mo(V) state [23,79,80]. It is unfortunate that the UV/visible absorption spectrum of the putative sulfido form of the enzyme, expressed and purified under anaerobic conditions, has not been reported since substitution of a Mo≡S for a Mo≡O group would be expected to significantly perturb the information-rich spectrum.

A final group of enzymes in the DMSO reductase family that have been extensively studied by EPR are the formate dehydrogenases, molybdenum- (and occasionally tungsten-) containing enzymes that catalyze the reversible interconversion of formate and CO_2_. As with the nitrate reductases, multiple varieties of formate dehydrogenase are known [81]. The FdnGHI and FdoGHI systems are trimeric, membrane-integral complexes that, with the NarGHI and NarYWV systems, respectively, contribute to a transmembrane potential owing to the fact that the membrane orientation for the Fdn enzymes is opposite to the Nar systems, with the subunit containing the active site molybdenum center being oriented toward the periplasm rather than the cytosol [70]. Next is the FdhF formate dehydrogenase from, e.g., *E. coli*, which is a dissociable, periplasmically localized subunit of the formate:H_2_ lyase complex [82]. Whereas the Fdn/Fdo (and Nar) systems possess multiple additional redox-active centers, FdhF is monomeric with molybdenum and [4Fe-4S] centers. Finally, there is the cytosolic FdsDABG enzyme from organisms such as *Cupriavidus necator* (formerly *Ralstonia eutropha*) [83,84,85] or *R. capsulatus* [86,87] that has an overall topology closely resembling the matrix-exposed portion of the mitochondrial Complex I [81,88]. These considerable structural variations and subcellular localization notwithstanding, the molybdenum-containing subunits/domains have a high degree of structural homology to one another and all possess a catalytically essential terminal Mo=S ligand [89]. *E. coli* Fdn/FdoGHI and FdhF have selenocysteine as the molybdenum ligand contributed by the polypeptide, while FdsDABG has a cysteine residue instead (the FdhF from *Pelobacter atrosepticum* also has cysteine rather than selenocysteine coordinated to the molybdenum).

The FdhF from *E. coli* yields a strong Mo(V) EPR signal upon substrate reduction, with weak coupling to a solvent-exchangeable proton and, when ^77^Se is used, strong coupling to selenium consistent direct SeCys coordination to the molybdenum [90,91]. A similar signal, shown in Figure 3 bottom right, is seen with the Cys-containing FdsDABG from *C. necator* [92] and *D. desulfuricans* [93]. Reduction of the enzyme from either organism with ^2^H-labeled formate in H_2_O that is initially devoid of proton coupling, but which grows in with time [91,92], the conclusion being that the hydrogen of substrate formate is transferred directly to the molybdenum center. The generally accepted reaction mechanism involves direct hydride transfer to the Mo^VI^=S to give a Mo^IV^-SH [92], analogous to the mechanism of xanthine oxidase above (Figure 2). A similar hydride transfer mechanism likely applies to the molybdenum- and tungsten-containing formylmethanofuran dehydrogenases from methanogenic archaea [94,95,96].

## 4. X-ray Absorption Spectroscopy

Since its development in the 1970′s, XAS has made critical contributions to our understanding of metalloproteins, including those containing molybdenum, owing to its ability to provide high-resolution structural information of the metal’s first coordination shell (and under favorable conditions, beyond) with non-crystalline samples. The precision in metal-ligand distances is at least an order of magnitude greater than obtained with X-ray crystal structures of even 1 Å resolution or better. The method involves scanning through and beyond the X-ray absorption edge (most commonly the K-edge, ionizing 1s electrons from the absorbing nucleus into the continuum). The emerging photoelectron wave has a de Broglie wavelength related to the difference in energy of the incident X-ray and the ionization energy, and is back-scattered by atoms in the immediate environment of the absorbing nucleus. As the scan progresses beyond the absorption edge, the de Broglie wavelength becomes progressively shorter, setting up a pattern of alternately constructive and destructive interference in the photoelectron wave for each scattering nucleus—the extended X-ray absorption fine structure, EXAFS. Analysis of the fine structure provides information not only about the absorber-scatterer distance and the number of scatterers at a given distance but also the chemical nature of the scatterer: N and O ligands are readily distinguished from S/Cl ligands and these from Se. On the other hand, it is typically not possible to distinguish N from O, etc. 

### 4.1. The Molybdenum Insertase

XAS studies of the molybdenum insertase Cnx1 double variant S269D D274S has provided considerable insight into the mechanism of molybdenum cofactor (Moco) formation, with the insertion of Mo from molybdate representing the last step in Moco biosynthesis [97,98]. The Mo K-edge exhibits an intense pre-edge feature assignable as an “oxo-edge” transition whose importance lies in the fact that its intensity correlates with the number of oxo donor ligands bound to the Mo ion [99,100]. By comparison with a variety of small inorganic oxomolybdenum molecules, it has been inferred that Cnx1 arrested at the point immediately prior to insertion possesses a MoO_2_ structure, which in turn was used to determine a [(PDT)MoO_2_(OH)]^1−^ structure for the intermediate. The mechanism of molybdate insertion derived from these XAS studies proposes that an oxo ligand of molybdate is labilized by protonation, with the protons coming from the PDT thiols to yield a [(PDT)MoO_3_]^2−^ structure that is presumably protonated by a nearby amino acid to yield the more thermodynamically stable [(PDT)MoO_2_(OH)]^1−^ structure. The [(PDT)MoO_2_(OH)]^1−^ species is then poised for insertion into apoenzymes, with protonation of the hydroxyl ligand leading to the displacement of water by an amino acid donor, which subsequently binds to the Mo ion. 

### 4.2. Xanthine Oxidase and Related Enzymes

XAS studies of bovine xanthine oxidase provided the initial evidence that the molybdenum coordination sphere of oxidized enzyme contained a terminal Mo=O, with two types of sulfur at shorter (~2.1 Å) and longer (~2.4 Å) distances from the metal [101,102,103]; similar results were subsequently obtained with the xanthine dehydrogenase from chicken liver, along with evidence that the Mo-S distance lengthened significantly, suggesting protonation of a Mo^VI^=S to Mo^IV^-SH [104]. Subsequent X-ray crystal structures [32,105] confirmed a Mo=S in oxidized enzyme and established the longer Mo-S distances to be due to the enedithiolate sulfurs of the pyranopterin cofactor. Although it was originally suggested that the Mo=S occupied an apical position in the molybdenum coordination, MCD studies indicated an apical oxo and equatorial sulfido [106], with this geometric arrangement being key to enzymatic catalysis [107]. It is now clear from subsequent X-ray crystallographic work that the Mo=O is indeed apical and the Mo=S equatorial within a square-pyramidal coordination geometry. Reduction results not only in protonation of the Mo=S to Mo-SH but also strengthening of the Mo=O bond to Mo≡O (again, involving one σ and two π Mo-O bonds). Additionally, although originally assigned from the original crystallographic work to be a bound water molecule on the basis of the long Mo-O distance, the equatorial (and catalytically labile) oxygen has subsequently been shown by XAS to be a shorter Mo-OH at a distance of 1.97 Å, shortening further to 1.74 Å at high pH as the Mo-OH deprotonates [108].

### 4.3. Sulfite Oxidase and Related Enzymes

**Figure 5:**
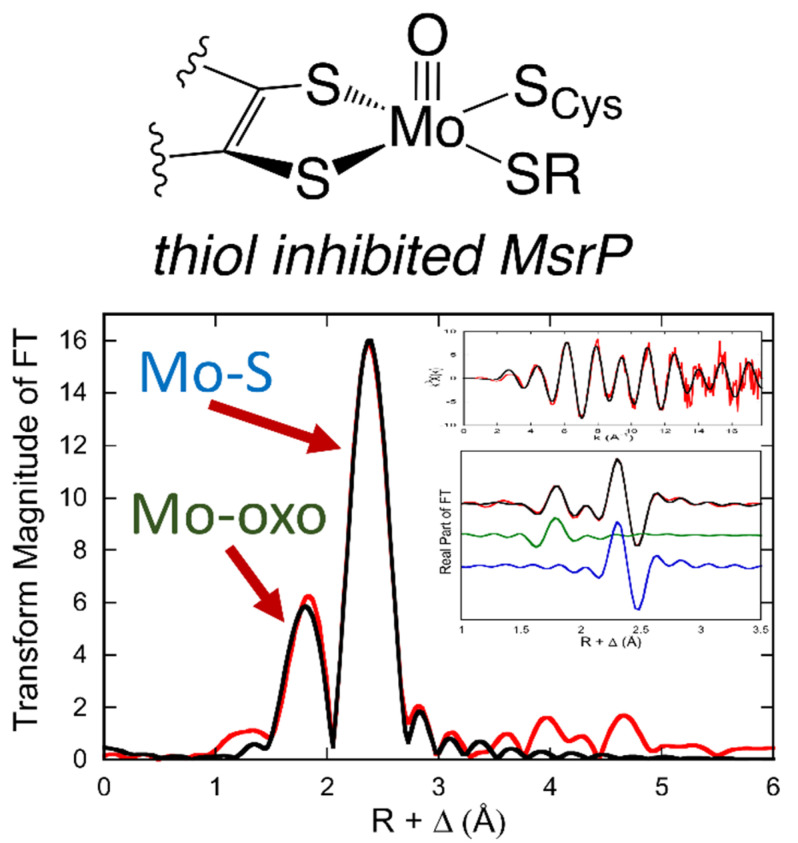
Mo K-edge XAS data for thiol inhibited MsrP. MsrP FT EXAFS data (red). Best fit to the data (black). Insets: EXAFS oscillations (top); real part of the FT (bottom). Data (red) and the best fit (black). Mo-oxo path is in green, and the Mo-S path is in blue. Adapted with permission from [19].

XAS studies also provided the initial evidence that the molybdenum coordination sphere of oxidized sulfite oxidase possessed two Mo=O groups as well as Mo-S, with one of the oxo groups becoming protonated upon reduction of the protein and the remaining bond being strengthened to Mo≡O [103,109,110]. Again, in the context of subsequent X-ray crystallography [111], the molybdenum coordination geometry in the oxidized enzyme is square-pyramidal, with an apical Mo=O, and an equatorial plane containing the second (catalytically labile) Mo=O, the two enedithiolate sulfurs of the pyranopterin cofactor and the sulfur of the coordinated cysteine. Human sulfite oxidase [111] and bacterial sulfite dehydrogenase [112] exhibit very similar EXAFS, as do the nitrate reductases from *A. thaliana* [113] and *Chlorella vulgaris* [114]. EXAFS has been used to probe the structure of the sulfite oxidase family proteins pmARC-1 (plant mitochondrial amidoxime reductase component) and HMCS-CT (the C-terminal domain of human molybdenum sulfurase, homologous to Cnx1 in plants) [115]. The data conclusively show that the latter is isolated in the reduced Mo(IV) state, leading to the proposal of an internal oxidation-reduction mechanism for Moco sulfuration by HMCS. EXAFS also indicates a novel thiol inhibited form of the Mo-dependent methionine sulfoxide reductase, MsrP (Figure 5) [19]. The derived [(PDT)MoO(S_Cys_)(S-R)] structure is supported by a combination of continuous-wave EPR, pulsed EPR, and model studies [19]. 

### 4.4. DMSO Reductase and Related Enzymes

The initial XAS analyses of members of the DMSO reductase family were, like the initial protein crystallography, hampered by structural heterogeneity at the molybdenum center. Again, this was ultimately shown in the case of the *R. sphaeroides* enzyme to be due to dissociation of one of the two pyranopterins (that designated Q crystallographically [67]) easily dissociated from the molybdenum, being replaced by a second Mo=O group [68]. It was eventually determined that redox-cycling the enzyme by reduction and reoxidation with substrate resulted in re-coordination of the Q pyranopterin [116], yielding a homogeneous sample. Subsequent XAS analysis of the redox-cycled *R. sphaeroides* enzyme is fully consistent with the oxidized molybdenum center being formulated as L_2_MoO(O-Ser) in a trigonal prismatic coordination geometry; full reduction results in protonation and dissociation of the Mo=O group, leaving an L_2_Mo(O-Ser) in a distorted square-pyramidal coordination geometry [117,118]. Similar results have been obtained with the *E. coli* DMSO reductase [119] and trimethylamine-N-oxide (TMAO) reductase [120], and *R. sphaeroides* biotin sulfoxide reductase [121]. As with DMSO reductase, one of the pyranopterins in TMAO reductase is readily dissociable but can be re-coordinated to the metal by redox-cycling [122]; this XAS work corrected the structure inferred from the crystal structure of the *Shewanella massilia* protein, which had been interpreted as L_2_MoO_2_(O-Ser)—i.e., seven-coordinate [122].

Given the extremely high level of accumulation of the catalytically relevant “high-g split” Mo(V) species in the course of turnover of the *R. sphaeroides* DMSO reductase with TMAO [20], this form of the enzyme has also been examined by XAS. It was found that a (presumably square-pyramidal) L_2_Mo(OH) structure with Ser 147 dissociated from the molybdenum best fit the data, although it was not possible to exclude an L_2_Mo(O-Ser) structure with the catalytically labile Mo=O (which was known to be transferred to a suitable acceptor in the course of turnover [123]) rather than the serine having dissociated from the molybdenum [124]. If this latter interpretation is correct, then reduction on to the Mo(IV) must involve the re-coordination of the serine, possibly explaining why this step is so slow. More recent EPR studies of 6-coordinate Mo(V) analogs [79] for the species giving rise to the “high-g split” EPR signal display EPR spin Hamiltonian parameters that are very similar to those determined for the enzyme [23], and support a hexacoordinate Mo(V) ion in the “high-g split” species with the O-Ser remaining coordinated to Mo. The EXAFS of these compounds can be equally well fit, however, with either a 5- or 6-coordinate model, highlighting the difficulty with XAS in determining the presence of one or two hydroxide/alkoxide ligands in the presence of multiple heavy atom donors bound to Mo. 

With regard to the FdhF formate dehydrogenase, the earliest XAS work was performed prior to the recognition that the functional enzyme possessed a Mo=S group. This played to one of the weaknesses of XAS, namely that it is often difficult to unambiguously determine the number of scattering nuclei at the same (or similar) distances, in this case between four and five sulfurs (one being at a shorter distance). Still, this early work demonstrated that selenocysteine was coordinated to the molybdenum in the *E. coli* FdhF [125]. More recently, the FdsDABG formate dehydrogenase from *R. capsulatus* has been examined (which has a cysteine ligand to the molybdenum rather than the selenocysteine seen in FdhF), and a short Mo=S is found in inverse proportion to a short Mo=O in comparing enzyme from one preparation to another [126]. This is most likely due to variations in the amount of nonfunctional enzyme that has lost the catalytically essential Mo=S.

## 5. Resonance Raman Spectroscopy

**Figure 6:**
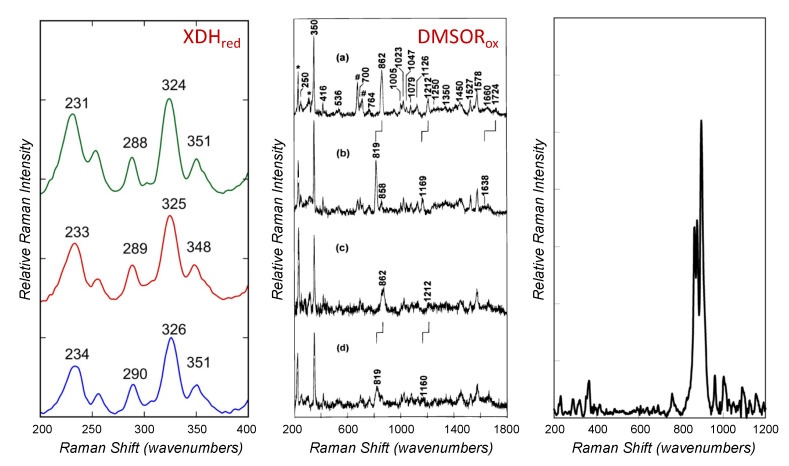
Resonance Raman spectra of xanthine dehydrogenase, sulfite oxidase and DMSO reductase. **Left**: Low-frequency resonance Raman spectra of Mo(IV)-product complexes formed from wt-XDH and specific variants using 4-thiolumazine as the substrate (green, Q197A; red, Q102G; blue, *wt*) [12]. Data were collected on resonance with the Mo(IV)−P MLCT band. **Middle**: *R. sphaeroides* DMSO reductase resonance Raman spectrum. (a) Dithionite-reduced enzyme re-oxidized with ^16^O DMSO; (b) dithionite-reduced enzyme reoxidized with ^18^O DMSO; (c) as prepared enzyme; (d) enzyme that was exchanged into ^18^O H_2_O buffer then dithionite-reduced and ferricyanide-oxidized. Lattice modes of ice (*), residual DMSO (#) Adapted with permission from [116]. **Right**: Resonance Raman spectrum of as-prepared plant SO from *A. thaliana*. Adapted with permission from [127].

Resonance Raman spectroscopy (Figure 6) involves irradiating a sample with a laser at a wavelength where the sample absorbs. In addition to normal Rayleigh scattering in which the incident photons are elastically scattered and emerge with the same energy as the incident beam, a fraction of the photons will excite vibrations within the sample and the scattered photon emerges with correspondingly less energy. Resonance Raman spectroscopy is thus intrinsically a vibrational phenomenon, although the selection rules are different than conventional infrared spectroscopy: in infrared spectroscopy, absorption occurs when the vibration causes a net change in dipole moment of the molecule, while in resonance Raman spectroscopy only vibrational modes that alter the polarizability along the transition dipole of the electronic transition are observed. 

### 5.1. Xanthine Oxidase and Related Enzymes

While the molybdenum center of xanthine oxidase does not contribute significantly to the UV/visible absorption spectrum of the enzyme, in the course of turnover with the pterin substrate lumazine two different long-wavelength absorbing charge-transfer complexes, corresponding to E_ox_•lumazine and E_red_•violapterin, are observed [10,128]. The complex of reduced enzyme with violapterin is particularly stable, with a fairly intense (ε = 8000 M^−1^cm^−1^) broad absorption band centered at ~650 nm, well isolated from the absorption of the enzyme’s FAD and iron-sulfur clusters. Upon excitation with 647-nm light, a variety of vibrational modes in the 600–1600 cm^−1^ range are observed, with those in the 1200–1600 cm^−1^ range being attributed to vibrational modes of the violapterin [128,129]. In agreement with subsequent crystallographic work with other substrates [32], it has been concluded that the violapterin must be coordinated directly to the molybdenum, presumably by the catalytically introduced hydroxyl group. Indeed, a mode at 1469 cm^−1^, downshifted to 1457 cm^−1^ when the sample was prepared in ^18^O-labeled water, has been attributed to a Mo-O-violapterin stretching mode. The only candidate mode for a Mo≡O stretch is at 853 cm^−1^, but it is downshifted only by 6 cm^−1^ to 847 cm^−1^ upon labeling with ^18^O, it being concluded that the Mo≡O stretch was not resonance-enhanced. Indeed, in light of subsequent crystallographic work it is evident that the charge-transfer transition dipole is in the molybdenum xy plane, with the Mo≡O along the z axis.

More recent resonance Raman studies on bovine xanthine oxidase and *Rhodobacter capsulatus* xanthine dehydrogenase (XDH) [11,12,13] used 4-thiolumazine and 2,4-dithiolumazine as reducing substrates to generate Mo(IV)-product complexes that possess a long wavelength MLCT band shifted to into the near-IR region of the optical spectrum. Raman bands in the 200–600 cm^−1^ region of the xanthine oxidase and XDH spectra are essentially identical, and the resonance Raman spectra for wild-type XDH and specific variant Mo(IV)-product complexes using 4-thiolumazine as the substrate are shown in Figure 6 left. The low-frequency vibrations observed in these studies were assigned as arising from Mo-PDT modes and product in-plane bending modes. Resonance enhancement of Mo-dithiolene core vibrations is consistent with the expected excited state distortions that accompany Mo(IV) → product charge transfer, which creates hole character on the Mo ion. Importantly, the creation of this transient hole character, coupled with the enhancement of Mo-ditholene vibrations, supports the PDT playing a critical role as an electron transfer conduit in the enzyme electron transfer half-reaction [12,13,130]. The near-IR MLCT band is assigned as a Mo(xy) → product π* transition with the product LUMO being the acceptor orbital in the transition. Thus, numerous high-frequency product vibrational modes are resonantly enhanced with excitation into this band since the Mo(xy) redox orbital is π-bonding with the product LUMO. The work indicated that excited state distortions occur within the Mo–O–C_product_ linkage, which represents a covalent pathway [8,107,131,132,133,134,135] for the two-electron oxidation of xanthine oxidase family substrates [11,36].

### 5.2. Sulfite Oxidase and Related Enzymes

The resonance Raman spectrum of the molybdenum center of the trypsin-cleaved molybdenum-containing domain of human sulfite oxidase (with an arginine engineered into position 108 [136]) has been examined, with good resonance-enhancement of vibrational modes using 488 nm excitation in the longest-wavelength absorption band of the domain [14]. Modes at 903 and 881 cm^−1^ shifted to 890 and 848 cm^−1^ upon redox-cycling the fragment in H_2_^18^O, and on the basis of comparisons with model compounds were assigned to symmetric and antisymmetric stretching modes, respectively, of the MoO_2_ unit with only one of the oxygens isotopically substituted under the conditions (a conclusion consistent with subsequent pulsed EPR studies indicating that the apical Mo=O group is not solvent-exchangeable [137]). Bands at 289 and 362 cm^−1^ seen with the wild-type fragment disappear in a C207S variant and were assigned to S-C and Mo-S stretching modes of the cysteine coordinated to the molybdenum. Bands at 419, 1006, 1161 and 1532 cm^−1^ seen with both the wild-type molybdenum-containing fragment and the C207S variant were assigned to specific modes of the enedithiolate of the pyranopterin cofactor. The resonance Raman results were used to tentatively assign the 480 nm absorption band in the enzyme as being primarily S_Cys_→Mo ligand-to-metal charge transfer transition (LMCT) in character. More recent resonance Raman studies on *A. thaliana* sulfite oxidase, [138] which lacks a *b*-type heme, confirm that the 20,833 cm^−1^ (480 nm) band is S_Cys_ → Mo LMCT in nature [14,138,139]. The higher energy (27,778 cm^−1^) charge transfer may be assigned as a S_dithiolene_→Mo LMCT transition [138]. Resonance Raman data collected at 30K using 488 nm excitation in resonance with the S_Cys_→Mo LMCT band showed resonance enhancement of bands at 896, 877, and 864 cm^−1^, in the Mo-dioxo stretching region of the spectrum. The assignment of these vibrations was significant and confirmed the remarkable inequivalence of the apical and equatorial oxo ligands in SO, which derive from the *trans* influence on the equatorial oxo ligand [138,139]. The importance of having a LUMO in SO that is primarily Mo(xy)–O_eq_ π* in nature is that the Mo(xy)–O_eq_ bond is selectively activated during oxygen atom transfer due to electron occupation of this orbital. As a result, the low-symmetry electronic structure of the enzyme translates into lowering the activation energy for atom transfer reactivity [138,139].

### 5.3. DMSO Reductase and Related Enzymes

The *R. sphaeroides* DMSO reductase has been the subject of the most extensive resonance Raman analyses. The initial resonance Raman work with the enzyme identified a 1575 cm^−1^ mode in oxidized enzyme that shifted to 1568 cm^−1^ upon reduction of the enzyme and was assigned to an enedithiolate C=C stretching mode [140]. Importantly, modes in the 330–380 cm^−1^ region were identified as Mo-S stretching modes from the metal-coordinated pyranopterin cofactor on the basis of their sensitivity to molybdenum oxidation state and ^34^S substitution. A comparison of the resonance Raman spectra of the enzyme with model compounds confirmed and quantified this latter assignment [141]. This work was performed prior to the recognition that DMSO reductase possessed two equivalents of the pyranopterin cofactor rather than just one, and with the crystal structure demonstrating that two equivalents of the cofactor were indeed present [68]. Subsequent resonance Raman work with oxidized, dithionite- and DMS-reduced, and glycol-inhibited enzyme explicitly took this into account [116]. In the oxidized enzyme, a single Mo=O stretch was identified at 862 cm^−1^ that shifts to 819 cm^−1^ when reduced enzyme is reoxidized with ^18^O-labeled DMSO; as expected, this mode was not evident in dithionite- or DMS-reduced sample, nor in glycol-inhibited enzyme. It was confirmed that modes in the 335–405 cm^−1^ range are due to various MoS_4_ modes of the two bound enedithiolate pyranopterins. Several enedithiolate modes in the 1000–1580 cm^−1^ region were also identified, including modes at 1527 and 1578 cm^−1^ that were assigned to the C=C stretch of two distinct enedithiolates, the first having a more π-delocalized electronic structure and the second having a more discrete dithiolate structure. 

## 6. Magnetic Circular Dichroism Spectroscopy

**Figure 7:**
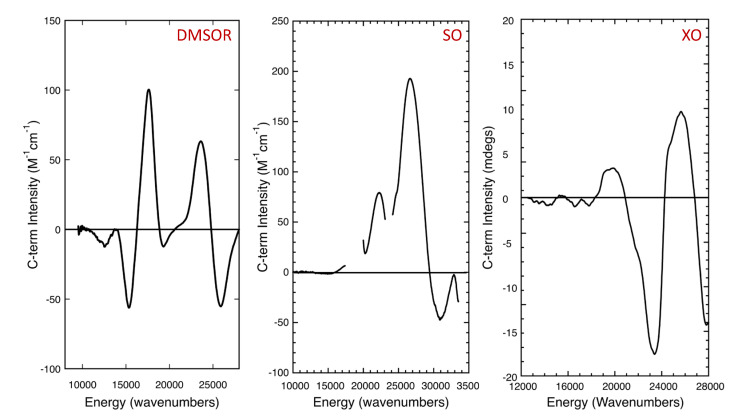
MCD spectra of DMSO reductase [23], sulfite oxidase [147], and xanthine oxidase [106]. **Left**: DMSOR “high-g split” collected under turnover conditions using TMAO as the oxidizing substrate, **Middle**: Chicken sulfite oxidase with b-heme contribution subtracted. **Right**: xanthine oxidase “very rapid” species generated using the slow substrate 2-hydroxy-6-methylpurine.

MCD spectroscopy is powerful probe of metalloenzyme active site electronic structure [142,143,144,145], and it has played an important role in enhancing our understanding of pyranopterin molybdenum enzyme active sites (Figure 7) [23,106,144,145,146,147]. MCD is complementary to EPR and electronic absorption spectroscopies and can selectively probe paramagnetic sites (e.g., Mo(V)) in the presence of a diamagnetic background to reveal electronic transitions at high resolution. Additionally, overlapping bands in electronic absorption spectra can often be resolved by using MCD spectroscopy since the MCD dispersion possesses sign. MCD requires the presence of spin-orbit coupling, and the intensity of MCD bands that originate from paramagnetic centers is expressed in terms of a C_0_/D_0_ ratio. Here, the temperature dependent MCD C-term intensity is given by C_0_ and the dipole strength of the transition by D_0_. These C_0_/D_0_ ratios are often largest for ligand field bands, since D_0_ is small for these transitions and the Mo spin-orbit coupling constant is large. The use of C_0_/D_0_ ratios can aid in the assignment of spectral features and variable-temperature MCD can be used to extract the paramagnetic C_0_-term from a strong diamagnetic background signal.

### 6.1. Xanthine Oxidase and Related Enzymes

Variable-temperature MCD spectroscopy has been used to probe bovine milk xanthine oxidase [106] (Figure 6, right). An apical sulfido ligand oriented *cis* to a terminal oxo donor was originally proposed for the active site of oxidized xanthine oxidase [105] and aldehyde oxidase [104] based on early X-ray crystallographic studies, but variable-temperature MCD spectroscopy on the enzyme was used to revise this original assignment. The MCD studies were performed on the Mo(V) “very rapid” intermediate seen with xanthine oxidase in the course of oxidizing 2-hydroxy-6-methylpurine, which can be generated under turnover conditions with the slow substrate 2-hydroxy-6-methylpurine. The MCD spectral features were assigned as deriving from a Mo(V) coordination geometry where the strong-field terminal oxo ligand is oriented *cis* to the PDT ene-1,2-dithiolate [106]. This relative orientation of the terminal oxo and the ene-1,2-dithiolate is also present in the model compound Tp*MoO(bdt), which had been studied previously by MCD spectroscopy [131]. Importantly, this geometry has been shown to enhance dithiolene S_ip_–Mo d(xy) covalency, and this covalency contribution has been suggested to contribute to an effective superexchange pathway for electron transfer regeneration of the oxidized active site, where electrons are shuttled sequentially from the reduced Mo center to the 2Fe-2S clusters and eventually to FAD. No MCD band was observed that could be assigned as a charge transfer transition involving the bound product, indicating that the product was either weakly bound to the Mo ion or the charge transfer band was outside of the experimental spectral window. The xanthine oxidase stereochemistry determined from the MCD studies were later confirmed by the X-ray structure of a reduced Mo(IV) intermediate that is formed using the slow substrate and mechanism-based inhibitor FYX051 [146].

### 6.2. Sulfite Oxidase and Related Enzymes

MCD spectroscopy has been used to probe the electronic structure of chicken sulfite oxidase that was poised in the catalytically relevant [Mo(V):Fe(II)] state [145,147] (Figure 6, middle). No charge transfer transitions were observed at energies below ~17,000 cm^−1^ and this was interpreted to result from a reduction in S_ip_–S_op_ orbital mixing and S_op_–Mo d(xy) covalency, in addition to a small dithiolene fold angle [130,145,147,148,149,150,151]. A band at 22,250 cm^−1^ was observed as a positive C-term and assigned as a S^σ^(cysteine)→Mo(xy) LMCT transition. This spectral assignment was used to conclude that the coordinated cysteine functions to reduce the effective nuclear charge of the Mo ion and move its reduction potential to more negative values via S→Mo charge donation. At higher energy MCD features were observed at 26,500 cm^−1^ and 31,000 cm^−1^, with the former being tentatively assigned as a S^op^(dithiolene)→Mo(xy) LMCT transition. A S^ip^(dithiolene)→Mo d(xy) transition was anticipated at ~19,000 cm^−1^ but this band was likely obscured by the Q-band MCD feature of the ferrous heme. MCD spectra have also been collected on the as-isolated Mo(V) form of MsrP [18], showing a strong correlation with observed electronic absorption bands. The results were initially interpreted in the context of an expected [(PDT)MoO(S_Cys_)(OH)]^1−^ structure, but more recent EXAFS, CW-EPR, pulsed EPR and model studies have been used to show that the Mo(V) from MsrP represents a thiol-inhibited species [19]. 

### 6.3. DMSO Reductase and Related Enzymes

Due to the lack of additional redox-active chromophores in the DMSO reductases from *R. sphaeroides* and *R. capsulatus*, these were the first mononuclear molybdenum enzymes to be studied by low-temperature MCD spectroscopy. The first MCD spectra were collected on a partially reduced Mo(V) enzyme form from *R. capsulatus*, which displayed hyperfine coupling to a single ^1^H nuclear spin [152]. The observed MCD was weak and the sample possessed only 0.06 spin/Mo, but this study nevertheless demonstrated that the MCD spectrum was comprised of positive and negative C-term MCD bands in the 300–800 nm range. In particular, the two oppositely signed C-term features in the 550–700 nm range (~14,300–18,200 cm^−1^) were assigned as arising from dithiolene→Mo(V) LMCT transitions. This assignment was based on a simple molecular orbital model and the assumption of *C_2v_* symmetry for the Mo-dithiolene fragment. The authors also stated that there was no spectral evidence for Mo-S_Cys_ coordination. A year later, data were collected on an inactive, glycerol inhibited, *R. sphaeroides* DMSO reductase sample with a markedly improved S/N ratio [153]. The data were qualitatively similar to that of the *R. capsulatus* enzyme and the analysis was similar, being based on the presence of only a single PDT coordinated to the Mo ion. Importantly, both of these early MCD studies provided strong support for Mo coordination to the dithiolene component of the PDT. Almost two decades later, a combined density function theory (DFT) and time-dependent DFT computational study was initiated to understand the electronic origin of the EPR spin-Hamiltonian parameters and the MCD spectrum of the glycerol inhibited, *R. sphaeroides* DMSO reductase enzyme [154]. A [Mo(PDT)_2_(OH)(OMe)]^1−^ computational model was used that incorporated hydrogen bonding from a histidine residue to the coordinated hydroxy ligand to reassign the original experimental MCD spectra [154]. This work was followed by a detailed spectroscopic and computational study on a bona fide Mo(V) catalytic intermediate in the *R. sphaeroides* enzyme [23]. With respect to the MCD spectrum of this intermediate (Figure 6, left) [23], a spectroscopically oriented configuration interaction (SORCI) method [155] was employed and band assignments were made for both the MCD and electronic absorption spectra in the 8000–28,000 cm^−1^ region of the spectra [23]. The relaxed active site geometry determined in this study was consistent with the EPR spin-Hamiltonian parameters, including a rhombic Mo A-tensor, and the MCD and electronic absorption spectra. The stability of this Mo(V) intermediate nicely accounted for enzyme reaction kinetics that show ~100% buildup of the intermediate under steady-state conditions using TMAO as the oxidizing substrate. The work was also important since it noted a reduced Mo-S_PDT_ covalency present in the redox orbital, which was directly probed by EPR spectroscopy, and this was suggested to slow the kinetics of reduction from the Mo(V) state to Mo(IV), as observed experimentally [23]. The spectroscopically determined geometry of the Mo(V) intermediate was also shown to be very similar to that of the transition state for oxygen atom transfer.

## 7. Conclusions and Prospectus

From our review of these spectroscopic studies, it should be abundantly clear that spectroscopic approaches have time and again provided critical insight into the structure and function of the mononuclear molybdenum enzymes, from the detailed geometry of the molybdenum center at the outset and completion of catalysis to the specific chemistry involved in the transformation of substrate to product. As new molybdenum-containing enzymes are discovered in the future, it is expected that spectroscopic methods such as those described here will continue to play critical roles in defining their geometric and electronic structures.
